# An extended window of opportunity for G-CSF treatment in cerebral ischemia

**DOI:** 10.1186/1741-7007-4-36

**Published:** 2006-10-18

**Authors:** Armin Schneider, Rainer Wysocki, Claudia Pitzer, Carola Krüger, Rico Laage, Stefan Schwab, Alfred Bach, Wolf-Rüdiger Schäbitz

**Affiliations:** 1 Axaron Bioscience AG, Im Neuenheimer Feld 515, 69120 Heidelberg, Germany; 2 Department of Neurology, University of Münster, Albert-Schweitzer-Str. 33, 48149 Münster, Germany; 3 Department of Neurology, University of Heidelberg, Im Neuenheimer Feld 400, 69120 Heidelberg, Germany; 4 Department of Neurology, University of Erlangen, Schwabachanlage 6, 91054 Erlangen, Germany

## Abstract

**Background:**

Granulocyte-colony stimulating factor (G-CSF) is known as a powerful regulator of white blood cell proliferation and differentiation in mammals. We, and others, have shown that G-CSF is effective in treating cerebral ischemia in rodents, both relating to infarct size as well as functional recovery. G-CSF and its receptor are expressed by neurons, and G-CSF regulates apoptosis and neurogenesis, providing a rational basis for its beneficial short- and long-term actions in ischemia. In addition, G-CSF may contribute to re-endothelialisation and arteriogenesis in the vasculature of the ischemic penumbra. In addition to these trophic effects, G-CSF is a potent neuroprotective factor reliably reducing infarct size in different stroke models.

**Results:**

Here, we have further delayed treatment and studied effects of G-CSF on infarct volume in the middle cerebral artery occlusion (MCAO) model and functional outcome in the cortical photothrombotic model. In the MCAO model, we applied a single dose of 60 μg/kg bodyweight G-CSF in rats 4 h after onset of ischemia. Infarct volume was determined 24 h after onset of ischemia. In the rat photothrombotic model, we applied 10 μg/kg bodyweight G-CSF daily for a period of 10 days starting either 24 or 72 h after induction of ischemia. G-CSF both decreased acute infarct volume in the MCAO model, and improved recovery in the photothrombotic model at delayed timepoints.

**Conclusion:**

These data further strengthen G-CSF's profile as a unique candidate stroke drug, and provide an experimental basis for application of G-CSF in the post-stroke recovery phase.

## Background

Granulocyte-colony stimulating factor (G-CSF) is a cytokine that stimulates generation of neutrophilic granulocytes, and has been in clinical use for more than 10 years in indications related to counteracting neutropenia, or for bone-marrow transplantations [1-3]. Recently, a surprising activity of G-CSF in protecting against experimental cerebral ischemia has been shown by a large number of laboratories [4-12]. The explanations for the effects of G-CSF on the CNS appear as a combination of direct anti-apoptotic activity on neurons [5,6,13] mediated by the presence of the cognate G-CSF receptor in a broad array of brain regions [14], stimulation of endogenous neurogenesis [14], regeneration of vascularisation [8], potential mobilisation of bone-marrow derived cells that migrate to the brain [11,12], and possible systemic anti-inflammatory effects ([15], discussed in [14]).

Characterised by this multimodal array, G-CSF appears beneficial for short-term damage parameters such as infarct size, and long-term effects on functional outcome (for example, see [6]). Based on this broad pre-clinical basis for efficacy, G-CSF is now undergoing testing in clinical trials by three different groups, primarily aimed at demonstrating safety in human stroke patients (reviewed in [16]).

Here we further explore the window of opportunity for G-CSF treatment in firstly, the most accepted model for evaluating neuroprotective effects, rat middle cerebral artery occlusion (MCAO), and secondly, the cortical photothrombotic stroke model for detecting effects on long-term functional outcome.

## Results

### Neuroprotective activity of G-CSF after delayed treatment

We recently reported an infarct-reducing capacity of G-CSF after middle cerebral artery occlusion (MCAO) when treatment was initiated at 30 min and 2 h after onset of ischemia [5,6]. Here we delayed treatment with G-CSF (60 μg/kg body weight i.v.) to 4 h after vessel occlusion. In addition, we analysed cortical and subcortical infarct volumes separately to determine relative protection levels in these areas. Infarct size was measured 24 h after induction of ischemia by TTC-staining. We obtained both direct infarct volumes (including edema volume) as well as indirect infarct volumes (contralateral minus ipsilateral non-infarcted volume) that do not contain edema under the assumption that edema formation is confined to infarcted areas. This also allowed us to deduct the edema volume for each animal.

**Figure 1:**
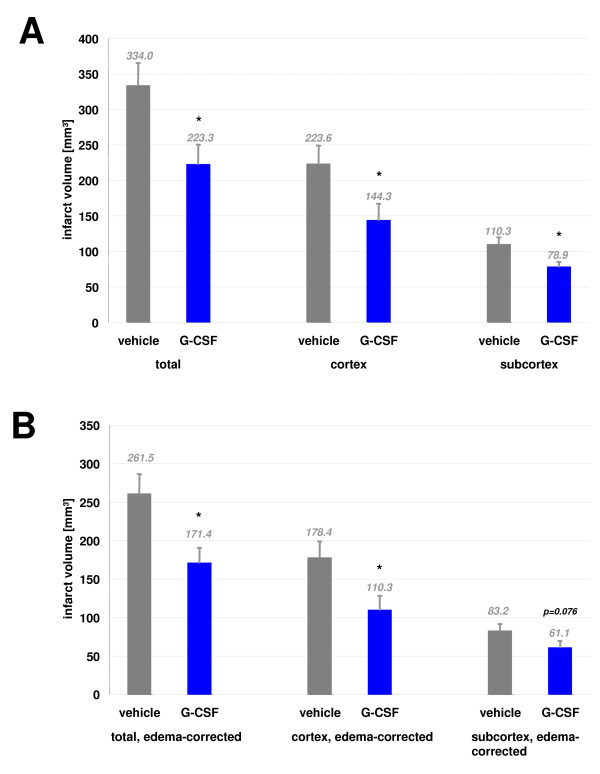
G-CSF reduces cortical and subcortical infarct volume in the rat MCAO model when applied 4 h after onset of ischemia. A. Shown are infarct volumes for the total infarct, cortical and subcortical areas. Infarct volumes were determined 24 h after onset of ischemia by TTC-staining and planimetry. B. Infarct volumes after edema correction. Edema-corrected infarct volumes were obtained by deducting the non-infarcted ipsilateral hemisphere volume from the contralateral hemisphere volume. Volumes are given in mm^3 ^as means ± SEM.

We observed a robust infarct volume reduction by 33 % in the total infarct volume as compared to vehicle-treated rats (334.0 ± 31.5 mm^3 ^(n = 24) vs. 223.3 ± 27.3 mm^3 ^(n = 18), p < 0.05; Figure 1A). This effect was significant in both the cortical (35% reduction; 223.6 ± 25.5 mm^3 ^vs. 144.3 ± 22.7 mm^3^; p < 0.05) as well as the subcortical (28% reduction; 110.3 ± 9.7 vs. 78.9 ± 6.3 mm^3^; p < 0.01) part of the infarction.

Relative infarct size reduction was equal after edema correction (34% reduction in total infarct size; 261.5 ± 24.9 mm^3 ^vs. 171.4 ± 19.4 mm^3^; p < 0.01, Fig. 1B), with 38% reduction in cortical volume (p < 0.05) and 27% in subcortical volume, which missed statistical significance (p = 0.076). There was no difference in mortality nor in several operational parameters obtained, such as oxygen saturation after the operation, the drop of cortical perfusion as monitored by Laser-Doppler flowmetry, or duration of the operation (data not shown).

### G-CSF improves functional outcome after cerebral ischemia

For measuring long-term effects of G-CSF treatment we applied photothrombotic induction of ischemia in the sensorimotor cortex, as this model produces defined neurological deficits without affecting survival. Previously we have shown a strong influence of G-CSF on functional outcome in the cortical photothrombotic model [6]. There, the first treatment was given 1 h after ischemia. To determine whether a more delayed initiation of treatment with G-CSF also induces a long-term sensorimotor functional improvement, we designed an experiment where G-CSF was given at 24 and 72 h after ischemia for 10consecutive days at a dose of 10 μg/kgbodyweight.

**Figure 2:**
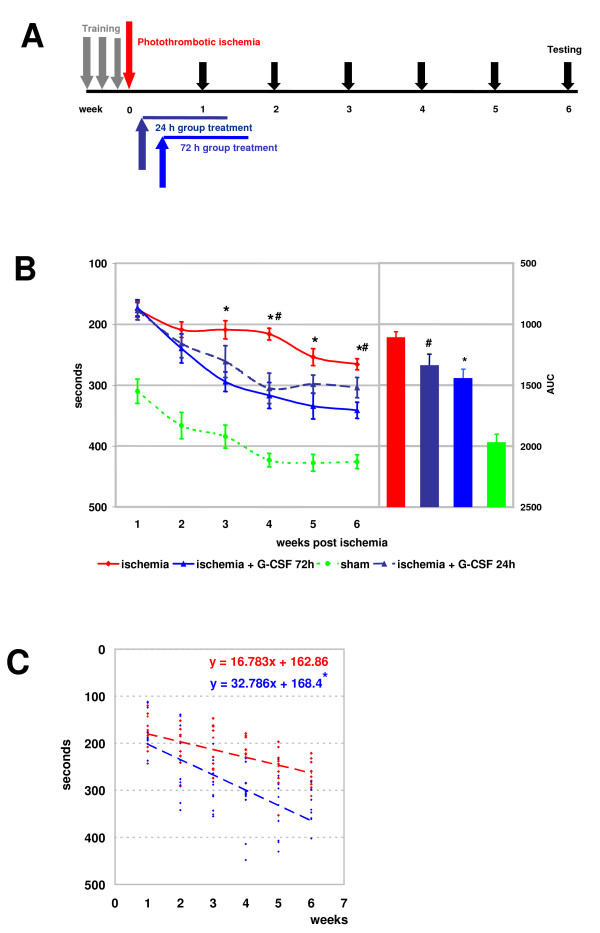
G-CSF improves functional outcome when given as late as 72 h after photothrombotic ischemia. A. Design of the cortical photothrombotic experiment. B. Rotarod performance over the course of 6 weeks after ischemia. Rats received 10 μg/kg bodyweight/day G-CSF for 10 days starting either at 24 or 72 h after ischemia. The effect is also detectable by an analysis of individual areas-under-the-curve (AUC). (*,#: p < 0.05 by ANOVA and Student-Newman-Keuls post-hoc test for 24 h (#) and 72 h (*) treatment). C. Linear regression analysis of vehicle treatment and G-CSF treatment initiated at 72 h demonstrates that slopes are significantly different by a factor of 2 (p = 0.004)

Sensorimotor deficits in the rotarod test were obvious in vehicle-treated ischemic animals compared to sham-operated rats in the rotarod for up to 6 weeks after the insult (Figure 2A). G-CSF treated ischemic rats in both the 24 h and 72 h time-window performed significantly better in the rotarod test than vehicle-treated animals when comparing group means per timepoint (Figure 2A). We also used another more appropriate test for time-series measurements in subjects, and compared areas under the individual curves over time (AUC) (Figure 1A, bar graphs). This data representation also demonstrated a robust improvement in sensorimotor function of G-CSF treatment over vehicle (ANOVA followed by SNK post hoc test; compare bars). There was no significant difference between the 24 and 72 h time window. Finally, we compared linear regressions of the vehicle-treated ischemia group, and the 72 h G-CSF treatment group (Figure 2B). This analysis demonstrated a significant difference in the slopes of both regression lines (p < 0.01), indicating a truly enhanced slope of recovery for the delayed G-CSF treatment. Physiological parameters (rectal temperature, pH, pCO_2_, pO_2_, and mean arterial pressure during surgery) did not differ between the groups. In addition, there were no differences in mortality or body weight between all groups (not shown).

## Discussion

### Infarct size reduction

Here we demonstrate a robust infarct-reducing effect of a 4 h treatment delay with G-CSF in a severe hemispheric stroke model (MCAO), viewed as the gold standard for testing neuroprotective effects. The end point for determining infarct volume was 24 h following onset of ischemia, a standard timepoint for neuroprotective studies. We know, however, that the effect of G-CSF is not transient as has been discussed with some experimental drugs, but is also detectable when examining the brain at 72 h post ischemia after a combined CCA/distal MCA occlusion [6]. The achieved total reduction in infarct volume of 34.5% is an extension of previous studies in the same model where treatment was initiated 30 min [5] and 2 h [6] after onset of ischemia. We also demonstrate for the first time a clear treatment effect both on cortical as well as subcortical areas with a meaningful sample size per group. This is important, as the effect of a number of neuroprotective drugs has been shown to be limited to the cortex, e.g. [17]. The neuroprotective acitivity of G-CSF in a broader range of brain regions can be explained by the broad distribution of the G-CSF receptor in the brain [6]. However, the effect on lesion volume in the cortex is more pronounced than in the subcortex, which is either explained by the relative distribution of infarct core and presumptive penumbra to striatum and cortex in the MCAO filament model, or indeed demonstrates a stronger protective effect in the cortex.

Support for a delayed window of opportunity for G-CSF in acute stroke models comes from three other independent studies. Treatment effects were investigated in comparatively mild ischemia models producing small hemispheric or cortical infarctions appropriate to assess recovery effects rather than to test the neuroprotective potential of a drug in the acute phase. Six et al. have shown a stunning 55% infarct reducing effect when treatment was delayed for 24 h after transient MCAO in the mouse with a relatively small sample size of n = 6 per group [4]. Komine-Kobayashi et al. demonstrated a decreased infarct size in the same model after initiation of G-CSF treatment 24 h and 72 h after the infarct [7]. Shyu et al. reported effects on infarct size when treatment was initiated as late as 24 h after the insult in a rat combined CCA/distal MCA model with 90 min occlusion of the distal MCA, which produces purely cortical infarcts [12]. Although these studies have used milder models, it appears probable from the cumulated evidence that the window of opportunity in a severe hemispheric stroke model could be also considerably longer than 4 h, especially in view of the still sizable treatment effect observed at this point. It is therefore desirable to further explore this acute time window and define the timepoint where efficacy is lost.

By comparing the differences of total infarct volumes without and with edema correction, our data indicate an absolute reduction in edema volume in favor for G-CSF (compare 51.9 mm^3 ^to 72.5 mm^3 ^in the vehicle treated group, Figure 1). This seems to be in agreement to the data generated by Gibson et al [10] who have seen edema reduction in both transient and permanent MCAO after G-CSF treatment. There, the authors have used a direct method to determine edema by measuring brain total water content by the dry weight method. However, as it is well known that larger infarcts produce more vasogenic edema, it is important to relate the edema volume to the infarct volumes in the respective groups to exclude edema reduction simply as a consequence of reduced infarct volume. Here, the edema volume is in similar proportion to the total infarct size in both the vehicle (21.7%) and the G-CSF group (23.2%). Thus, there is no evidence for a separate effect on edema extent by G-CSF treatment. As there are no data in the work mentioned above to estimate the relative edema extent to infarct sizes, which are decreased by G-CSF treatment, it is difficult at the moment to judge whether the data can be truly interpreted as an independent edema-reducing effect of G-CSF.

### Long-term effects

We have previously already demonstrated significant activity of G-CSF towards enhancing post-stroke recovery in the photothrombotic model in conjunction with stimulation of neurogenesis by using a diligent assessment of sensorimotor function [6]. In this previous experiment we have applied a five-day treatment of G-CSF at 15 μg/kg bodyweight/day starting with the first dose 1 h after induction of ischemia. Therefore it is possible that part of the recovery effect seen is due to the acute antiapoptotic property of G-CSF. To minimise any acute effects on infarct size, and define time windows for G-CSF's pro-regenerative functions after stroke, we delayed the initial treatment to 24 and 72 h post ischemia. In addition, we chose a dosage scheme that appears clinically safe and feasible for a treatment period of weeks, 10 days of 10 μg/kg bodyweight daily. This application scheme is similar to the one used in the previous recovery experiment, but has a longer duration and lower single doses of G-CSF. This scheme is also readily translatable to an explorative study in stroke patients for testing the pro-regenerative potential in man. For exploring the therapeutic time window for initiation of a recovery-enhancing effect post-stroke we have here concentrated on a robust parameter, time to fall off the rotarod device. The course of the curves for all ischemic groups neatly demonstrates that the groups only start to separate in the second week of the experiment, and highlight that the initial value post-stroke is absolutely identical, indicating that there was no difference in the extent of the initial damage in this model relevant to sensorimotor performance. Thus, we have reached our goal to uncouple acute neuroprotective effects of G-CSF from effects on long-term recovery.

Surprisingly, a recovery-enhancing effect at the 72 h delayed treatment initiation was still clearly detectable, and not significantly different from the initiation at 24 h. This indicates that the window of opportunity for pure recovery enhancement with G-CSF may not be time-dependent in the same way as therapeutic effects in the acute phase after stroke. Indeed, there is no statistical difference between the 24- and 72-h initiation of treatment. A substantial window of opportunity for improvements in functional outcome by G-CSF treatment is also suggested by Lees et al. [8], who report effects in rotarod measurements particularly when treatment was delayed to 1 day and longer after MCAO.

It will now be very interesting to further delay treatment and to define the limits of G-CSF efficacy in such models. There is a surprising scarcity of data relating to the characterisation of susceptible post-stroke time periods for recovery processes in the rodent. In light of the neurogenesis-enhancing effects of G-CSF it is highly interesting that, at least for ischemia-induced neurogenesis, there is no clear time dependence from the insult, and increased neurogenesis may keep up for months after stroke [18], thereby likely providing a long period of susceptibility to therapeutic interference by neurogenesis-enhancing substances such as G-CSF.

### Implications for the clinic

Here, we have further delineated a truly bimodal efficacy of G-CSF both for acute effects on infarct size, as well as on chronic enhancement of post-stroke recovery. Together with a substantial body of evidence for efficacy of G-CSF in varied stroke models (reviewed in [14]) our data further strengthen confidence in the potential of G-CSF for the treatment of human stroke.

In addition to a rational mode-of-action within stroke pathophysiology, any candidate for further stroke drug development should be explored according to the STAIR criteria [19]. G-CSF fulfills these criteria well: blood-brain-barrier penetration, neuroprotective activity in different stroke models including permanent ischemia demonstrated by independent groups, activity shown in different species, well-known pharmakocinetics, as well as functional outcome data. Likewise, data on the potential therapeutic time window are important. We have recently shown that G-CSF is neuroprotective until at least 2 h post-stroke in a severe focal cerebral ischemia model [6]. As indicated by the present results, G-CSF is effective when treatment is delayed as late as 4 h post-stroke. Although conversions to the human situation are delicate, a 4-h time window in this stroke model may relate to a therapeutic time window in humans of several hours.

The effect on functional outcome appears as a separable activity of G-CSF, independent of its neuroprotective potential. This makes G-CSF a highly attractive candidate for recovery enhancement in the subacute or chronic phase of a stroke and increases the likelihood for detecting clinical outcome improvement in acute stroke trials.

## Conclusion

Aiming at the two principal modes-of-action of G-CSF in the brain, antiapoptosis and regeneration enhancement, we have both studied effects on infarct size with delayed treatment in a severe MCAO model, as well as functional sensorimotor outcome in the cortical photothrombotic model. We find that G-CSF has an acute time window of at least 4 h post-stroke, and an astonishing time window for effects on functional outcome of three days after stroke. The strong effects on functional outcome distuinguish G-CSF from most other candidate stroke drugs in development.

Our findings further strengthen confidence in the efficacy of G-CSF both for infarct size reduction, and improvement of functional parameters, and are important guiding lines for future clinical development.

## Methods

### Suture occlusion model

Male Wistar rats weighing 280 to 320 g received inhalation anesthesia with 70%N_2_O, 30% O_2_, and 1% halothane. The right femoral vein was cannulated and used for drug delivery. During the experiment, core body temperature was monitored and maintained at 37°C by a thermostatically controlled heating pad (Föhr Medical Intruments). Middle cerebral artery occlusion (MCAO) was induced with a silicon-coated (Provil Novo, Heraus Kulzer) 4-0 nylon filament (Ethicon) that was introduced into the common carotid artery and advanced into the internal carotid artery as described previously [5]. Successful MCA occlusion was verified by Laser-Doppler flowmetry (Perimed 4000) with a probe positioned over the cortical MCA territory. After 90 min MCAO, the filament was withdrawn to allow for reperfusion. Four hours after onset of occlusion animals received 60 μg/kg G-CSF (Neupogen, AMGEN) i.v. over 20 min or vehicle.

### Infarct and edema volume

Infarct volumes were determined 24 h after induction of ischemia by TTC staining as described previously [5]. 2 mm sections were cut using a brain matrix (Harvard Apparatus, Inc.), and stained with 2,3,5-Triphenyl tetrazolium chloride (TTC, Sigma-Aldrich) for 10 min at 37°C. Stained sections were scanned on both sides using a color scanner, and infarct areas determined using ImageJ v 1.33 [20]. "Direct" infarct volume was obtained by integrating measured infarcted areas, "indirect" infarct volume was obtained by deducting the non-infarcted volume of the infarcted hemisphere from the contralateral hemisphere. As edema mostly develops in the infarcted areas, the "direct" volume contains edema, and "direct" minus "indirect" volume gives the edema volume (see also [5]). Animals with any signs of subarachnoid hemorrhage (barrel-rolling, or blood in the ventricles or subarachnoid space), any cortical damage from drill holes, and no or minimal infarcts on TTC-staining were excluded from the analysis before unblinding.

### Photothrombotic ischemia model

Male Wistar rats were anesthetised with an intramuscular injection of 100 mg/kg bodyweight ketaminehydrochloride (Ketanest) and 8 mg/kg bodyweight xylazine hydrochloride (Rompun). Anesthesia was maintained with 50 mg/kg bodyweight ketaminehydrochloride and 4 mg/kg bodyweight xylazine hydrochlorideif necessary. A PE-50 polyethylene tube was inserted into the right femoral artery for continuous monitoring of mean arterial blood pressure, and blood gases. The right femoral vein was cannulated by a PE-50 tube for treatment infusion. During the experiment rectal temperature was monitored and maintained at 37°C by a thermostatically controlled heating pad (Föhr Medical Intruments). Photothrombotic ischemia was induced in the rat parietal cortex according to the method of Watson et al. [21]. Animals were placed in a stereotaxic frame, and the scalp was incised for exposure of the skull surface. For illumination, a fiber-optic bundle with a 1.5-mm aperture was placed stereotaxically onto the skull 4 mm posterior to the bregma and 4 mm lateral from the midline. The skull was illuminated with a cold, white light beam (150 W) for 20 minutes. During the first 2 min of illumination, the dye rose bengal (0.133 mL/kg body weight, 10 mg/mL saline) was injected intravenously. Sham-operated animals underwent the same experimental procedures as described above without infusion of rose bengal and illumination. After surgery, the catheters were removed, and the animals were allowed to recover from the anesthesia and given food and water ad libitum. For treatment, ischemic or sham-operated animals were given vehicle (n = 10) or 10 μg G-CSF/kg bodyweight i.v. starting 24 (n = 10) or 72 hours (n = 10) after induction of ischemia. Daily repeated i.v. bolus infusions via the tail vein (G-CSF or vehicle) followed on the following 10 days.

### Behavioral measurements

In all animals Rotarod testing was performed during the light cycle before ischemia after a training period of 3 days, and at 1,2,3,4,5 and 6 weeks after ischemia by an investigator blinded to the experimental groups. Rats were placed on an accelerating Rotarod cylinder (increased speed from 4 to 40 rpm within 5 min). The time the animals remained on the Rotarod was measured, and the trial ended if the animal fell off the rungs or gripped the device and spun around for two consecutive revolutions without attempting to walk on the rungs. An arbitrary time limit of 500 sec was set for the rats on the Rotarod cylinder in training and in the testing procedures. The animals were trained 3 days before ischemia, and the mean duration (sec) on the device was recorded with three measurements.

All animal experiments were performed in accordance with national and international regulations, and were approved by the appropriate government authorities. All experiments were performed in a fully randomised and blinded fashion.

### Data and Statistical analysis

The values presented in this study are means ± SEM. The two-tailed t-test was used to determine significant difference of infarct volumes. Behavioural measurements were analysed using ANOVA and the Student-Newman-Keuls post hoc test. Area under the curve (AUC) was calculated using the trapezoidal algorithm. Linear regressions were compared using coincidence testing. Statistics were performed with NCSS 2004 (NCSS Statistical Software) or Primer of Biostatistics software. A p value <0.05 was considered statistically significant.

## Authors' contributions

RW, CP, CK, RL carried out the animal studies, and participated in the data analysis. AS and WRS carried out data analyses. AS, WRS, SS, AB conceived of the study, and participated in its design and coordination and helped to draft the manuscript. All authors read and approved the final manuscript.
